# Sphingomyelinase decreases transepithelial anion secretion in airway epithelial cells in part by inhibiting CFTR‐mediated apical conductance

**DOI:** 10.14814/phy2.14928

**Published:** 2021-08-12

**Authors:** Kirsten A. Cottrill, Raven J. Peterson, Colby F. Lewallen, Michael Koval, Robert J. Bridges, Nael A. McCarty

**Affiliations:** ^1^ Molecular and Systems Pharmacology PhD Program Emory University Atlanta Georgia USA; ^2^ Biochemistry, Cell, and Developmental Biology PhD Program Emory University Atlanta Georgia USA; ^3^ Georgia Institute of Technology G.W. Woodruff School of Mechanical Engineering Atlanta Georgia USA; ^4^ Division of Pulmonary, Allergy, Critical Care and Sleep Medicine Department of Medicine Emory University Atlanta Georgia USA; ^5^ Department of Cell Biology Emory University Atlanta Georgia USA; ^6^ Department of Physiology and Biophysics Center for Genetic Diseases Chicago Medical School North Chicago, Illinois USA; ^7^ Department of Pediatrics and Children’s Healthcare of Atlanta Center for Cystic Fibrosis and Airways Disease Research Emory University School of Medicine Atlanta Georgia USA

**Keywords:** ceramide, conductance, cystic fibrosis, cystic fibrosis transmembrane conductance regulator, electrophysiology, epithelial cell, sphingomyelinase, tight junction

## Abstract

The cystic fibrosis transmembrane conductance regulator (CFTR) is an anion channel whose dysfunction causes cystic fibrosis (CF). The loss of CFTR function in pulmonary epithelial cells causes surface dehydration, mucus build‐up, inflammation, and bacterial infections that lead to lung failure. Little has been done to evaluate the effects of lipid perturbation on CFTR activity, despite CFTR residing in the plasma membrane. This work focuses on the acute effects of sphingomyelinase (SMase), a bacterial virulence factor secreted by CF relevant airway bacteria which degrades sphingomyelin into ceramide and phosphocholine, on the electrical circuitry of pulmonary epithelial monolayers. We report that basolateral SMase decreases CFTR‐mediated transepithelial anion secretion in both primary bronchial and tracheal epithelial cells from explant tissue, with current CFTR modulators unable to rescue this effect. Focusing on primary cells, we took a holistic ion homeostasis approach to determine a cause for reduced anion secretion following SMase treatment. Using impedance analysis, we determined that basolateral SMase inhibits apical and basolateral conductance in non‐CF primary cells without affecting paracellular permeability. In CF primary airway cells, correction with clinically relevant CFTR modulators did not prevent SMase‐mediated inhibition of CFTR currents. Furthermore, SMase was found to inhibit only apical conductance in these cells. Future work should determine the mechanism for SMase‐mediated inhibition of CFTR currents, and further explore the clinical relevance of SMase and sphingolipid imbalances.

## Graphical abstract

The loss of function of cystic fibrosis transmembrane conductance regulator (CFTR), in people with cystic fibrosis (CF), causes airway surface dehydration, mucus build‐up, inflammation, and bacterial infections that lead to lung failure. This work evaluates the effects of lipid perturbation on CFTR activity, focusing on the acute effects of sphingomyelinase (SMase), a bacterial virulence factor secreted by CF‐relevant airway bacteria, on the electrical circuitry of pulmonary epithelial monolayers. We report that basolateral SMase decreases CFTR‐mediated transepithelial anion secretion in both primary bronchial and tracheal epithelial cells from explant tissue, with current CFTR modulators unable to rescue this effect.

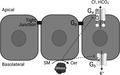

## 1 INTRODUCTION

The cystic fibrosis transmembrane conductance regulator (CFTR) is a chloride and bicarbonate anion channel present in epithelial cells throughout the human body. CFTR, located at the apical side of airway epithelial cells, is activated by phosphorylation by the cAMP‐activated protein kinase A (PKA), and is gated by the binding and hydrolysis of ATP (Berger et al., 1991; Frizzell & Hanrahan, 2012). Loss‐of‐function mutations in CFTR cause the lethal genetic disease cystic fibrosis (CF) (Riordan et al., 1989; Rommens et al., 1989). Most commonly, people living with CF die of pulmonary failure caused by dehydration of the airspace, chronic inflammation, bronchiectasis, mucus buildup, and chronic lung infections (Cystic Fibrosis Foundation, 2018; Elborn, 2016). While some therapeutics have been developed that target the primary CFTR defect, these therapeutics do not fully recover CFTR channel function (Guimbellot et al., 2017; Joshi et al., 2019). A better understanding of how CFTR functions in the pulmonary epithelial cell environment is necessary for the development of more efficacious treatment strategies for CF and other lung diseases.

Membrane proteins such as CFTR depend on their membrane lipid environment, a fact that is often neglected experimentally due to difficulties in controlling and examining this environment. It is possible that a perturbation of the cellular lipid environment could affect the function of CFTR, as has been seen in other ion channels (Bao et al., 2007; Hill et al., 2007; Levitan et al., 2010). Specifically, we and others have previously found that the bacterial virulence factor sphingomyelinase (SMase), an enzyme which breaks down membrane‐localized sphingomyelin into ceramide and phosphocholine, has an inhibitory effect on CFTR‐mediated transepithelial currents in airway epithelial cells (Ito et al., 2004; Stauffer et al., 2017). We also found that the only clinically approved CFTR potentiator, VX770/Ivacaftor, did not rescue this loss of channel activity in clinically relevant primary airway epithelial cells both from non‐CF and CF donors (Stauffer et al., 2017). The precise mechanism by which SMase decreases these transepithelial currents is not known at present.

Sphingolipids are involved in regulation of cell physiology, including cell growth and apoptosis, among other things (Maceyka & Spiegel, 2014; Spiegel & Merrill, 1996), and their roles in inflammatory lung diseases have been reviewed in more detail elsewhere (Maceyka & Spiegel, 2014). Understanding the sphingolipid imbalance caused by SMase is clinically relevant to many chronic lung diseases. It has been found in bronchial epithelial cells that oxidative stress, present in many inflammatory lung diseases (Dua et al., 2019; Vliet et al., 2018), increases the activity of endogenous neutral‐SMase (Chan & Goldkorn, 2000; Lavrentiadou et al., 2001). Furthermore, proinflammatory factors such as tumor necrosis factor α and lipopolysaccharide promote the secretion and activation of acid‐SMase from lung epithelial cells (Jenkins et al., 2010; Kornhuber et al., 2015). However, endogenous SMase is not the only source of this enzyme. The two bacteria most commonly found in the CF lung, *Pseudomonas aeruginosa* and *Staphylococcus aureus* (Cystic Fibrosis Foundation, 2018), both secrete enzymes with SMase activity (Barker et al., 2004; Huseby et al., 2007).

**FIGURE 1:**
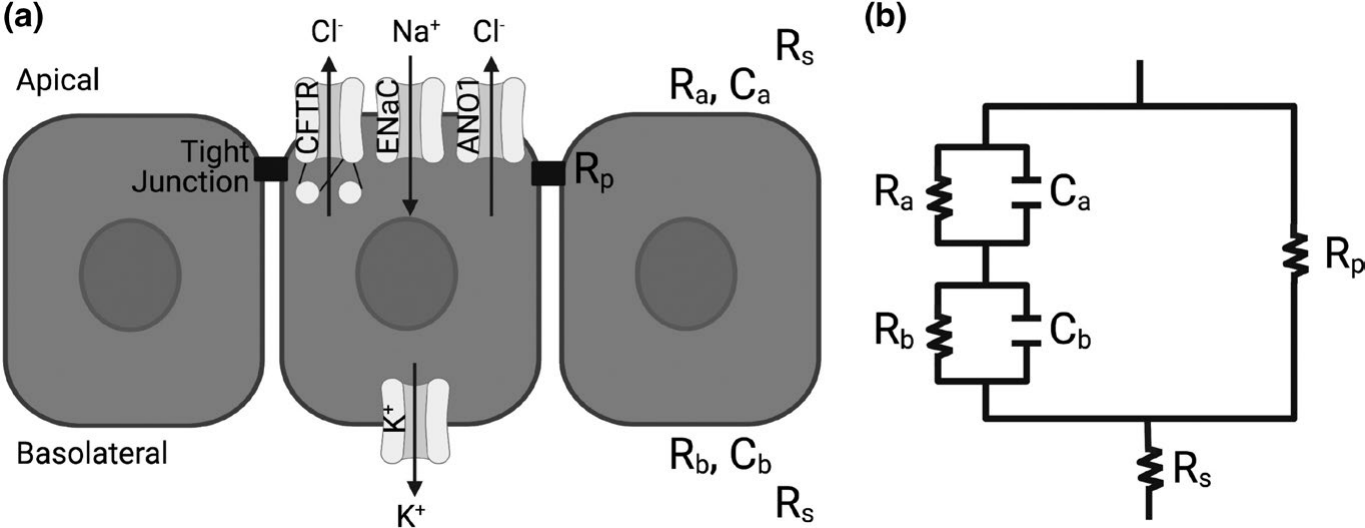
A model of a bronchial epithelial monolayer (a) is shown alongside the representative circuit diagram (b). The cells are gray rectangles, the channels are light gray, the tight junctions are black rectangles, and the directionalities of ion flow are black arrows. The apical and basolateral capacitances are *C*_a_ and *C*_b_, respectively. The apical resistance (*R*_a_) is affected by apical channels CFTR, epithelial sodium channel (ENaC), and anoctamin 1 (ANO1). The basolateral resistance (*R*_b_) is affected by channels such as the basolateral potassium (K^+^) channel. The paracellular resistance (*R*_p_) is due to tight junctions between the cells. The series resistance (*R*_s_) is driven by the composition of the buffer and the resistance of the plastic filter on which the cells are supported. The apical resistor and capacitor are in parallel with one another, as are the basolateral resistor and capacitor. The apical components are in series with the basolateral components. The paracellular resistor is in parallel with both the apical and basolateral components, while the solution resistor is in series with the entire circuit. This figure was made using BioRender

Given the relevance of these sphingolipid imbalances, more work was needed to fully understand the mechanism of SMase‐mediated inhibition of transepithelial CFTR currents. A monolayer of bronchial epithelial cells has many components that contribute to transepithelial currents (Figure 1). Decreased transepithelial anion currents such as observed after treatment with SMase could be due to a loss of chloride or bicarbonate conductivity through the apical side of the cell, a loss in the driving force facilitated by ion channels on the basolateral side of the cell, a change in the paracellular shunt pathway, or some combination of these. Furthermore, it is unknown whether this mechanism is consistent between non‐CF human bronchial epithelial cells (nHBEs) and CF human bronchial epithelial cells (cfHBEs), and whether it is a robust effect that is observed across cells from many donors or from other regions of the lung. The goal of the work presented here was to understand in more detail the mechanism by which SMase decreased transepithelial anion currents in primary airway epithelial cells.

## 2 MATERIALS AND METHODS

Unless otherwise specified, chemical reagents were purchased from MilliporeSigma.

### 2.1 Airway epithelial cells from human donors

Two different methods for preparing and culturing primary airway epithelial cells were used. One of these methods is based on work from the Randell laboratory (Fulcher et al., 2005), and has been used previously in our laboratory (Stauffer et al., 2017). Primary bronchial (HBE), tracheal (HTE), and mixed bronchial/tracheal airway (HAE) cells were isolated from human donor lung explants under an IRB‐approved protocol through the CF@LANTA Experimental Models Core. Cells were plated on Costar 3470 plates (0.4 μM pore size, polyester, Corning), and after 2 days were transitioned to air‐liquid interface (ALI) and differentiated in medium based on the previously described ALI medium formulation (Fulcher et al., 2005), with modifications to glucose (150 mg/dl; 8.3 mM), CaCl_2_ (1 mM), heparin (2 µg/ml), l‐glutamine (2.5 mM), hydrocortisone 960 mg/ml, bovine pituitary extract (20 µg/ml), and Mg^2+^ (0.5 µM). Once cultures were at ALI, the medium was changed every 48–72 h and cultures were allowed to differentiate for at least 3 weeks. For cfHBEs, which were from donors homozygous for the ΔF508 mutation, CFTR was corrected either by temperature shift to 27°C accompanied by addition of 3 µM VX809 (Selleckchem) for 24 h, or by treatment with 18 µM VX661 (Selleckchem) and 5 µM VX445 (Selleckchem) for 48 h.

Impedance measurement experiments described below were conducted using nHBEs and cfHBEs which were cultured using the Vertex method (Neuberger et al., 2011). The HBE cells were plated on Costar 24‐well high‐throughput screening filter plates (0.4 μM pore size, polyester, Corning) and grown at ALI in HBE differentiation medium containing 2% Ultroser‐G for 5–7 weeks. Medium was replaced on the basolateral side three times a week. To remove accumulated mucus, the apical surface was washed with 70 µl of freshly made 3 mM DTT in PBS for 30 min. Cells were washed 72 h later with 70 µl of PBS for 30 min, 24 h before the functional measurements. For impedance measurements, specifically, trafficking of ΔF508‐CFTR in cfHBEs was corrected with 6 µM C18 (VRT‐534, an analog of VX809) for 24 h prior to the experiment (Eckford et al., 2014).

### 2.2 Calu‐3 and 16HBE bronchial epithelial cell culture

For Ussing Chamber experiments, Calu‐3 cells (ATCC^®^ HTB‐55^™^) were grown as described previously (Ito et al., 2004). Cells were plated at 150,000 cells/well on Costar 3470 plates using DMEM medium with 10% FBS (Atlanta Biologicals), 2 mM l‐glutamine (Corning), 50 IU/ml penicillin (Corning), and 50 μg/ml streptomycin (Corning). The apical medium was removed 1 day after plating, and the cells were allowed to continue to polarize for 5–7 days. The basolateral medium was replaced every 48–72 h during this polarization period.

16HBEs were grown similar to Calu‐3s, except that MEM medium (Gibco) was used as a base and the cells were grown in submersion culture on Transwells for 5–7 days.

### 2.3 Purification of bacterial SMase

Wild‐type (WT) and enzyme‐dead H322A *S*. *aureus* SMase with an N‐terminal 6x histidine tag were purified from BL21 (DE3) *Escherichia coli* bacteria by recombinant expression from a pET28b vector (Novagen) similar to previous methods (Stauffer et al., 2017). Bacteria were grown with 50 µg/ml kanamycin sulfate (Thermo Fisher), induced with 0.1 mM IPTG, and incubated at 16°C overnight. Bacteria were pelleted, then resuspended in PBS (Boston BioProducts) with 1:100 protease inhibitor cocktail. Bacteria were lysed by sonication, and insoluble material was pelleted at 11,000 *g* for 30 min at 4°C. The soluble material (supernatant) was incubated overnight at 4°C with Roche cOmplete^™^ resin, washed, and equilibrated according to the manufacturer's protocol. The resin was washed five times with wash buffer at 4°C. Resin was incubated for 30 min at 4°C in three column volumes of elution buffer. This elution fraction was collected, and an additional three column volumes of elution buffer was added to the beads for 5 min at 4°C. These elution fractions were pooled, diluted to 4 mg/ml, and dialyzed in 1 L wash buffer in 12–24 h increments using Spectrum Labs Float‐A‐Lyzers^®^ with 0.1 kDa molecular weight cutoff until the calculated concentration of imidazole was below 1 nM. SMase purity was evaluated by a Coomassie‐Blue‐stained SDS‐PAGE. Final protein concentration was determined by a Thermo Scientific Pierce^™^ BCA assay kit in accordance with the manufacturer's protocol. Specific activity of 100 ng of SMase protein at room temperature over 30–40 min was determined by a Molecular Probes^®^ Amplex^®^ Red SMase activity assay in accordance with the manufacturer's protocol, using a plate reader (SpectraMax^®^ M2e, Molecular Devices). Note that SMase is added basolaterally in this study, as we and others have shown that WT SMase has no effect when applied apically (Stauffer et al., 2017). We have shown that WT SMase does not hydrolyze apical sphingomyelin (Stauffer et al., 2017).

### 2.4 Lipidomics mass spectrometry

All chemical reagents in this section are from Thermo Fisher, unless otherwise noted. Polarized HBEs were collected from Transwell filters by scraping with a pipet tip, pelleted at 500 *g* for 10 min, flash‐frozen with liquid nitrogen, and stored at −80°C. Pellets were resuspended in 100 μL isopropyl alcohol and subjected to three freeze‐thaw cycles using liquid nitrogen and ice bath sonication. After each extraction, samples were dried in a SpeedVac vacuum concentrator and reconstituted with the reconstitution solvent, detailed below, at a ratio of 3.33E‐4 μL/cell. Reconstituted samples were sonicated for 5 min and centrifuged at 21,100 *g* for 5 min. The supernatant was transferred to autosampler vials, sealed and stored at 4°C until analysis. For quality control purposes, a pooled sample was created by mixing an equal volume from each sample. A sample blank was created by following the above procedure without cells.

Ultra‐performance liquid chromatography coupled to mass spectrometry (UPLC‐MS) was performed using a Vanquish system, an Accucore^™^ C30 column (2.1 × 150 mm, 2.6 µm particle size), and an Orbitrap ID‐X Tribrid mass spectrometer system. The chromatographic method for sample analysis involved elution with solutions including: Mobile Phase A (40:60 water:acetonitrile, 10 mM ammonium formate, 0.1% formic acid); Mobile Phase B (10:90 acetonitrile:isopropyl alcohol [A461‐4], 10 mM ammonium formate, 0.1% formic acid). The following gradient program was used at a flow rate of 0.40 ml/min: 0 min 80% A, 1 min 40% A, 5 min 30% A, 5.5 min 15% A, 8 min 10% A, held 8.2 min to 10.5 min 0% A, 10.7 min 80% A, and held until 12 min. The column temperature was 50°C, and the injection volume was 5 µl. The heated electrospray ionization source was operated at a vaporizer temperature of 275°C, spray voltage of 3.5 kV and sheath, auxiliary, and sweep gas flows of 40, 8, and 1, respectively. The instrument acquired full MS data in the 150–2000 m/z range in positive ionization mode. UPLC‐MS/MS experiments were performed by acquiring mass spectra in a data‐dependent fashion. MS/MS were collected with a resolution of 120,000 and the dd‐MS2 were collected at a resolution of 30,000, an isolation window of 0.8 m/z, and a cycle time of 2 s. Stepped normalized collision energies of 15, 30, and 45 were used to fragment selected precursors in the HCD cell prior to combination of ion for analysis in the orbitrap. Dynamic exclusion was set at 2.5 s. Ions with charges >2 were omitted.

Data acquisition and processing were carried out using Compound Discoverer^™^ V3.0. After processing, peak areas were scaled by the median peak area of the sample. Annotation of the dataset was achieved by MS2 spectral matching to a local spectral database, built from curated experimental data. In addition, accurate mass, retention time, and isotopic pattern were matched to database entries.

Standards were obtained from Avanti Polar Lipids, Inc., including the following, listed with their final target concentration diluted in Optima^™^ chloroform: 160 µg/ml 15:0–18:1(d7); 5 µg/ml 15:0–18:1(d7) PE; 5 µg/ml 15:0–18:1(d7) PS; 30 µg/ml 15:0–18:1(d7) PG; 10 µg/ml 15:0–18:1(d7) PI; 25 µg/ml 18:1(d7) LPC; 5 µg/ml 18:1(d7) LPE; 350 µg/ml 18:1(d7) Chol Ester; 10 µg/ml 15:0–18:1(d7) DG; 55 µg/ml 15:0–18:1(d7)‐15:0 TG; 30 µg/ml 18:1(d9) SM; 100 µg/ml cholesterol (d7). The reconstitution solution was prepared by diluting the stock solution and Deuterated Ceramide LIPIDOMIX^™^ Mass Spec Standard (d18:1‐d7/16:0, d18:1‐d7/18:0, d18:1‐d7/24:0, and d18:1‐d7/24:1(15Z)) to 1.66% and 0.22% v/v, respectively, in isopropanol.

Peak areas were normalized to a blank tube, lipid standards, and the median peak intensity of all identified features. Data are reported either in bar graphs with the normalized peak area on the *y*‐axis, or as volcano plots with the −log (unadjusted *p* value) on the *y*‐axis and the difference in the normalized peak intensities on the *x*‐axis. A 5% false discovery rate was applied using the two‐stage step‐up method of Benjamini et al. (2006), as recommended by PRISM. Statistical values were considered significant if both the *p* value and the *Q* value were <0.05.

### 2.5 Short‐circuit current measurements

Short‐circuit current experiments using an Ussing Chamber were performed as described previously (Cui et al., 2019; Stauffer et al., 2017). A VCC MC6 multichannel voltage/current clamp with a U100 converter, EasyMount containment systems, P2300 chambers, DM MC6 input modules, P2024‐40 electrode leads, P2020‐S electrode sets, P2020 electrode tips, and P2302T Transwell sliders were obtained from Physiologic Instruments. Acquire & Analyze software was used to collect data. The amplifier was set to voltage‐clamp at 0 mV to record short‐circuit currents.

Bath buffer was prepared according to the recipe described previously (Ito et al., 2004). Normal Chloride Buffer was 115 mM NaCl, 5 mM KCl, 1 mM MgCl_2_, 2 mM CaCl_2_, 10 mM glucose, 10 mM HEPES, 25 mM NaHCO_3_, and pH 7.4. low chloride buffer was 115 mM Na gluconate, 5 mM KCl, 1 mM MgCl_2_, 4 mM CaCl_2_, 10 mM glucose, 10 mM HEPES, 25 mM NaHCO_3_, and pH 7.4. In most cases, a chloride gradient was applied by adding the normal chloride buffer basolaterally and the low chloride buffer apically. Chambers were bubbled with O_2_:CO_2_ 95:5% and maintained at 37°C during experiments.

Cells were stabilized for 30 min prior to treatment with 1 µg/ml WT or H322A (inactive mutant) SMase basolaterally for 30 min. To inhibit ENaC‐mediated sodium currents, 20 µM amiloride was added to the apical side of cells. To generate cAMP to stimulate CFTR, 0.01–10 µM forskolin was added to both sides of the cells. To potentiate CFTR currents, 1 µM VX770 (Selleckchem) was added to both sides of the cells. To inhibit CFTR, 10 µM INH172 was added to the apical side of cells. The currents elicited by each treatment were calculated as the average of the final 10 s prior to the next treatment.

### 2.6 Impedance measurements

Impedance analysis experiments were performed as described previously (Singh et al., 2002; Tamada et al., 2001). For 1 h prior to experiments, cells were switched to HEPES‐buffered (pH 7.4) F12 Coon's modified medium at 37°C without CO_2_. This plate was transferred to a 37°C plate warmer on an automated robotic platform. Transepithelial voltage (*V*_T_) and resistance (*R*_T_) of all 24 wells were measured simultaneously every 3 min using a MTECC24 (Multi Transepithelial Current Clamp [EP Design]). A reference plate in a separate 37°C plate warmer on the same robotic platform was used to measure the offset potential of the electrodes between measurements of the cell plate. Impedance was measured every 3 min at 126 frequencies ranging from 1 to 10,000 Hz, fitting only the 1–8000 Hz range. Cells were stabilized for 15 min prior to basolateral addition of 1 µg/ml WT or H322A SMase. After 30 min of SMase treatment, at least 6 µM benzamil was added apically to inhibit epithelial sodium channels (ENaC). After 18 min, 10 µM forskolin and 1 µM VX770 were added to activate and potentiate CFTR currents. The values are reported after 30 min of addition of forskolin and VX770.

A monolayer of epithelial cells was modeled as in Figure 1. The impedance data were plotted as Nyquist diagrams. Custom software, developed in the Bridges Laboratory (Singh et al., 2002), was used to fit these Nyquist plots to solve for the apical and basolateral resistances and capacitances (*R*_a_, *R*_b_, *C*_a_, *C*_b_), using a system of equations described previously (Margineanu & Van Driessche, 1990). In order to fit these equations to the Nyquist plot, an initial estimate value for the paracellular resistance (*R*_p_), the inverse of paracellular conductance (*G*_p_), is needed. Many current‐voltage relationship experiments performed previously in the Bridges laboratory revealed that in nHBEs and cfHBEs, following the addition of benzamil to inhibit ENaC and forskolin/VX770 to activate CFTR, *G*_p_ is 71% of the transepithelial conductance (*G*_T_) (data not shown).

Lastly, it must be assumed that the basolateral membrane time constant (*τ*_b_), equal to the product of resistance and capacitance, is larger than the apical membrane time constant (*τ*_a_). Microelectrode experiments have determined that basolateral resistance is larger (Kreindler et al., 2005; Tamada et al., 2001). Furthermore, since membrane capacitance is based on membrane surface area, thickness, and lipid composition, and since the basolateral membrane has a significantly larger surface area than the apical membrane, it is safe to assume that the basolateral capacitance is larger than the apical capacitance (Gentet et al., 2000). These considerations, in addition to pharmacological studies targeting the apical and the basolateral membrane, indicate that *τ*_b_ is larger than *τ*_a_ (Tamada et al., 2001).

With all of the assumptions discussed here, the software can successfully iteratively solve for the values of *R*_a_, *R*_b_, *C*_a_, and *C*_b_ (within certain reasonable bounds) to determine the best fit for the impedance data. These best‐fit values are reported within this manuscript.

### 2.7 Calcein flux assay

Paracellular flux was measured using calcein (Schlingmann et al., 2016; Stewart et al., 2017). Krebs Ringers HEPES (KRH) buffer was 150 mM NaCl, 2 mM CaCl_2_, 1 mM MgCl_2_, 10 mM glucose, 10 mM HEPES, and pH 7.4. Cells were washed once with KRH, then left at 37°C in fresh KRH buffer, with or without 3 mM EGTA to disrupt tight junctions for 90 min. To the basolateral side of the cells, 500 µl of fresh KRH buffer (with or without 3 mM EGTA) with 1 µg/ml H322A or WT SMase was added. From this, 100 µl were immediately collected as the 0 min time point, and a fresh 100 µl with the appropriate SMase treatment was added back to the basolateral side. To the apical side, 200 µl of KRH with 20 µg/ml calcein (Invitrogen) was added. Cells were maintained at 37°C, and 100 µl of basolateral buffer was collected and replaced every 15 min over the course of 1 h. Collected basolateral buffer was maintained in a black well, clear bottom, Costar 96‐well plate protected from light. After all time points were collected, the 96‐well plate was read with a Spectromax^®^ M2e plate reader with excitation and emission wavelengths of 485 and 515 nm, respectively, to determine the relative transepithelial flux of calcein.

### 2.8 Statistical analyses

Data were exported to and processed in Excel, unless otherwise noted. Statistical analyses were conducted and graphs were made using GraphPad PRISM software, with α set at 0.05 according to common practice. Data were excluded based on Grubb's outlier tests or in cases of technical experimental issues. In all cases, data are represented as the mean and standard deviation, unless otherwise noted. In many cases, the individual data points are also plotted. Specific statistical tests as well as the significance values are listed in the figure legends, including unpaired, two‐tailed *t*‐tests; multiple *t*‐tests without correction; and two‐way ANOVAs, utilizing the Tukey correction recommended by PRISM if multiple comparisons were used.

## 3 RESULTS

### 3.1 WT SMase affects the sphingolipid profile in nHBEs

**FIGURE 2:**
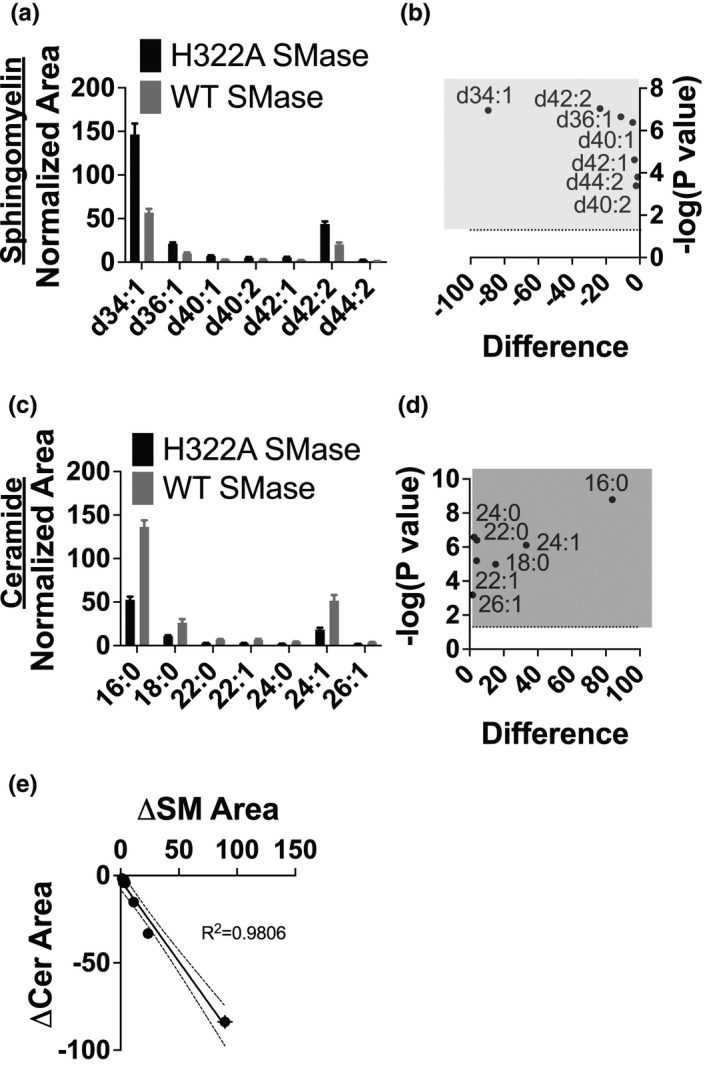
Lipidomics mass spectrometry revealed (a, b) a reduction of sphingomyelins (SM) and (c, d) an increase of ceramides in nHBEs (a single biological replicate is shown) treated basolaterally with WT SMase (gray, *n* = 5), as compared to enzyme‐dead H322A SMase control (black, *n* = 6). (a, c) The means and standard deviations of the peak amplitudes (normalized as described in Methods) are shown. Data were analyzed by multiple *t*‐tests, applying a 5% false discovery rate as described in Methods. (b, d) Volcano plots of the differences between the WT Mase and H322A SMase control treatments, with the –log(*p* value) on the *y*‐axis and the difference in the normalized peak areas on the *x*‐axis, are shown. The horizontal dotted line (‐log(*p* value) = 1.30) indicates the significance cutoff for the *p* value, above which a species is considered significantly different so long as the *Q* value was also <0.05. All sphingomyelins of interest were significantly decreased by WT SMase treatment (within the light gray box). Conversely, all ceramides of interest were significantly increased (within the dark gray box). (e) The mean and SEM of the change in sphingomyelin was plotted against the mean and SEM of the change in the probable corresponding ceramide. A linear fit was applied and indicated high correlation between these two axes (*R* ^2^ = 0.9806)

As a control for our WT SMase treatment, enzyme‐dead H322A SMase was purified in parallel to account for any co‐purified proteins. The WT and H322A SMase preparations appear nearly identical in purity, in which SMase is by far the most dominant band (Figure S1a). H322A SMase was confirmed to have no detectible SMase activity (Figure S1b). In order to determine the sphingolipid profile of polarized nHBEs, and how that profile changes with SMase treatment, cells were treated basolaterally with 1 µg/ml WT SMase or H322A SMase control for 30 min prior to extraction of cell lipids for analysis by lipidomics mass spectrometry (Stauffer et al., 2017). Preliminary experiments on polarized Calu‐3 bronchial epithelial cells were performed to determine if a simple isopropyl alcohol extraction was as efficient at extracting sphingolipids of interest as the ethyl acetate method previously outlined (Bielawski et al., 2006; Guilbault et al., 2008). In fact, we found that isopropyl alcohol was more efficient than ethyl acetate at extracting sphingomyelins and ceramides, and thus this method was used for the remainder of the experiments. (Figure S2). Only sphingolipids confirmed at a Schymanski confidence level 3 or higher were assessed (Figure 2) (Schymanski et al., 2014). The highest abundance sphingomyelins identified in nHBEs both with H322A and WT SMase treatment were the d34:1, d42:2, and d36:1 species. Our mass spectrometry instrumentation did not allow for the distinction between the sphingosine tail and the fatty acid chain of the sphingomyelins studied. However, the highest abundance ceramide species (d18:1/16:0, d18:1/24:1, and d18:1/18:0) likely correspond to the highest abundance sphingomyelin species. This hypothesis is supported by correlating the decrease in sphingomyelin species to the increase in the corresponding ceramides after WT SMase treatment, which results in a 0.9806 *R* ^2^ value (Figure 2e). The relative abundance of these three species of ceramides in bronchial epithelial cells generated by WT SMase was similar, but not identical, to previous studies from other labs (Garic et al., 2017; Petrache et al., 2005; Zehethofer et al., 2015). In nHBEs, WT SMase treatment decreased the abundance of all sphingomyelin species by 54.3 ± 2.3% (SEM) while increasing all ceramide species by 144.6 ± 9.6% (SEM). Because WT SMase broadly decreased all sphingomyelins and increased all ceramides, we could not parse out any specific sphingolipid interactions caused by WT SMase treatment. Note also that WT SMase added to the apical side of bronchial epithelial cells elicits no effect (Ito et al., 2004; Stauffer et al., 2017). We have found previously that, for an as yet undetermined reason, WT SMase does not hydrolyze sphingomyelin on the apical side of the cells (Stauffer et al., 2017).

### 3.2 WT SMase decreases short‐circuit CFTR current in nHBEs and nHTEs

We have shown previously that WT Mase added to the basolateral side of mixed bronchial/tracheal airway epithelial cells (nHAEs) reduces forskolin‐elicited and VX770‐potentiated transepithelial short‐circuit currents, defined as the change in current from the post‐amiloride current to the post‐forskolin or post‐VX770 current (Stauffer et al., 2017). To further understand if these differences were due to a change in current through CFTR, we quantified the difference in the current sensitive to INH172, which inhibits CFTR channels (Ma et al., 2002). INH172 also has been found to inhibit the volume‐sensitive outwardly rectifying chloride channel, but this channel is not expected to be activated by forskolin (Melis et al., 2014). Furthermore, we used INH172 at concentrations and exposure times that avoid pitfalls related to generation of reactive oxygen species and mitochondrial dysfunction (Kelly et al., 2010). Thus, we have defined CFTR currents within this study as forskolin‐elicited, VX770‐potentiated, and INH172‐sensitive transepithelial short‐circuit currents.

**FIGURE 3:**
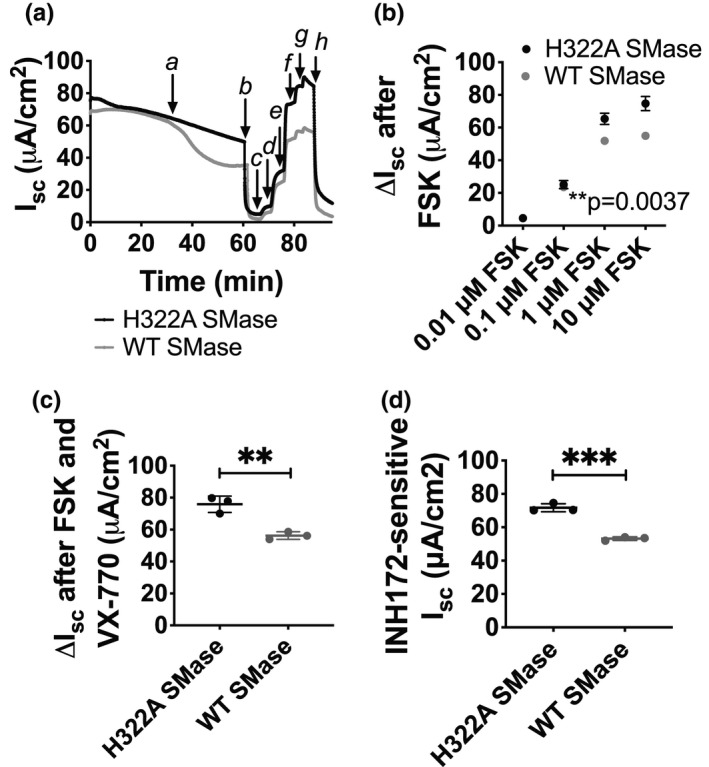
Short‐circuit current analysis revealed a reduction of transepithelial CFTR current in nHBEs after WT SMase treatment. A single biological replicate is shown. (a) An example trace of area‐corrected transepithelial short‐circuit currents is shown. Cells were stabilized for 30 min, at which point (a) 1 µg/ml enzyme‐dead H322A (black, *n* = 3) or WT SMase (gray, *n* = 3) was added basolaterally. After 30 min, (b) 20 µM amiloride was added apically, followed by (c–f) 0.01, 0.1, 1, and 10 µM forskolin. Forskolin‐elicited currents were potentiated by (g) 1 µM VX770. Finally, CFTR currents were specifically inhibited by (h) 10 µM INH172 apically. (b) The changes between the post‐amiloride current and the current elicited by various concentrations of forskolin were analyzed by a two‐way ANOVA with repeated measures over the concentrations of forskolin. This analysis indicates that WT SMase significantly decreased forskolin‐elicited currents in nHBEs (**p = 0.0037). The changes in current (c) from the post‐amiloride current to the post‐VX770 current and (d) from the post‐VX770 current to the post‐INH172 current were analyzed by unpaired two‐tailed *t*‐tests. These analyses indicate that WT SMase significantly decreased the VX770‐potentiated (**p = 0.0038) and INH172‐sensitive (***p = 0.0003) currents in nHBEs

Because of a refinement of our isolation techniques, we were able to evaluate bronchial and tracheal epithelial cells separately. Thus, in order to understand if WT SMase had a more dominant effect on bronchial or tracheal epithelial cells, we repeated our previous experiments in both. A representative experiment is shown (Figure 3a). Consistent with our previous work in nHAEs, we found that WT SMase treatment of nHBEs reduced forskolin‐elicited currents (Figure 3b). VX770‐potentiated currents also were reduced by WT SMase treatment from 75.8 ± 5.1 to 56.3 ± 2.3 µA/cm^2^, an approximately 25.8% decrease (Figure 3c). Similarly, WT SMase significantly decreased INH172‐sensitive current from 71.7 ± 2.4 to 53.1 ± 1.1 µA/cm^2^, (Figure 3d), a 25.9% decrease further indicating that CFTR currents were indeed reduced by WT SMase. This effect of WT SMase on forskolin‐elicited and INH172‐sensitive currents was found in nHBEs from multiple donors (within this study, each figure represents a different unique subject, while the data within a figure represents technical replicates from that subject), indicating that this is not a patient‐specific phenomenon. In nHTEs, the forskolin‐elicited, VX770‐potentiated, and INH172‐sensitive currents also were found to be decreased by WT SMase treatment (Figure S3), indicating that WT SMase broadly affects CFTR‐mediated anion secretion in multiple classes of airway epithelial cells. Note also that the effects of WT SMase on CFTR currents in nHBEs remained consistent when the short‐circuit current analyses were performed under symmetric chloride conditions (data not shown).

The forskolin‐elicited and VX770‐potentiated current data were calculated as the change in current from the post‐amiloride current. The INH172‐sensitive current was normalized similarly from the post‐VX770 current to the post‐INH172 current. To confirm that these reduced forskolin‐elicited, VX770‐potentiated, and INH172‐sensitive currents were not due to an increase in the post‐amiloride or post‐INH172 baselines currents, we quantified the absolute currents after amiloride and after INH172. These baseline currents were not significantly different after WT SMase treatment compared to control (Figure S4a,d). However, the absolute currents following forskolin addition were decreased by WT SMase (Figure S4c). This indicates that WT SMase specifically decreased currents through CFTR in nHBEs. Interestingly, along with decreasing CFTR currents, WT SMase decreased ENaC‐mediated currents, as indicated by the reduction in amiloride‐sensitive current (Figure S4b). This phenomenon has been seen by others, for example when WT SMase and ceramide were shown to decrease ENaC open probability in renal cells (Bao et al., 2007). However, it cannot be ruled out that this decrease in ENaC current is at least partially due to decreased activity of basolateral potassium channels that facilitate the potential for sodium movement through ENaC.

### 3.3 Differential effect of WT SMase on immortalized Calu‐3 and 16HBEs

**FIGURE 4:**
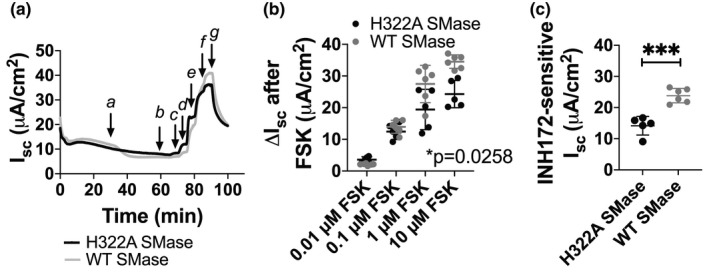
Short‐circuit current analysis revealed an increase in CFTR currents in Calu‐3s after WT SMase treatment. (a) An example trace of area‐corrected transepithelial short‐circuit currents is shown. Cells were treated as in Figure 3, except VX770 was not added prior to (g) INH172 addition. (b) The forskolin‐elicited current was analyzed by a two‐way ANOVA with repeated measures over the concentrations of forskolin, indicating that WT SMase (gray, *n* = 6) significantly increased forskolin‐elicited currents as compared to H322A SMase control (black, *n* = 5) in Calu‐3s (*p = 0.0258). (c) INH172‐sensitive currents were analyzed by an unpaired two‐tailed *t*‐test, indicating that WT SMase significantly increased the INH172‐sensitive currents in Calu‐3s (***p = 0.0003)

Previous work had reported that WT SMase decreased forskolin‐elicited currents in the immortalized bronchial epithelial cell line Calu‐3 under symmetric chloride conditions (Ito et al., 2004). We thus repeated our previous short‐circuit current analyses on Calu‐3s. In contrast to previous results, under a chloride gradient, Calu‐3 bronchial epithelial cells did not replicate the phenotype seen in nHBEs following WT SMase treatment. Rather, we found that WT SMase increased CFTR currents in Calu‐3s, as indicated by increased forskolin‐elicited and INH172‐sensitive currents (Figure 4). This is the opposite of the decreases in CFTR currents seen in nHBEs. We repeated our Calu‐3 experiments in symmetric chloride conditions to exactly replicate the experimental setup in previous literature. However, we found that while WT SMase had no significant effect on forskolin‐elicited currents, it actually increased the INH172‐sensitive currents similar to the experiments performed under a chloride gradient (data not shown). The reasons for the inconsistency of our results as compared to previous literature are unknown at present, but could reflect a significant heterogeneity to the Calu‐3 cell line. Importantly, the previous analysis did not use a CFTR‐specific inhibitor such as INH172 to verify that the currents were attributable to CFTR, instead using the non‐specific inhibitor NPPB (Ito et al., 2004).

We then asked whether the immortalized 16HBE bronchial epithelial cell line was a suitable surrogate for nHBEs. Interestingly, WT SMase treatment caused an elevated post‐amiloride baseline current (Figure S5b). Given that the post‐forskolin currents were not different between treatments, the apparent decrease in forskolin‐elicited currents following WT SMase treatment was likely due to this elevated post‐amiloride current (Figure S5c,d). Interestingly, the post‐INH172 currents were not affected by WT SMase, nor were the INH172‐sensitive currents. Taken together, this suggests that WT SMase actually activates CFTR prior to forskolin addition. To test this hypothesis, we treated 16HBEs with INH172 prior to amiloride to determine if the elevated post‐amiloride currents remained in the SMase‐treated cells. This INH172 pre‐treatment did eliminate the elevated post‐amiloride currents in the WT SMase‐treated cells, suggesting that in 16HBEs, WT SMase treatment activates CFTR prior to forskolin addition (Figure S5g,h). This is different than what occurs in both the nHBEs and the Calu‐3s, suggesting that the effects of WT SMase are not consistent between cell types. Given that nHBEs are a closer in vitro analog to native cells in vivo, we chose to perform all future experiments in nHBEs.

### 3.4 WT SMase affects impedance‐derived apical and basolateral conductance in nHBEs

The results thus far establish that CFTR‐mediated transepithelial short‐circuit currents in nHBEs are reduced by WT SMase treatment. However, transepithelial currents are controlled by basolateral and apical conductance of ions through channels, along with functionally related transporters, and paracellular conductance through tight junctions (Figure 1). It is important to note that conductance describes the ability for ions to move through a channel or across a membrane, independent of the net ion movement, which is current. In order to understand if basolateral WT SMase reduced transepithelial currents by inhibiting CFTR‐mediated apical membrane conductance, by inhibiting basolateral conductance, or both, we utilized impedance analysis, which estimates apical and basolateral resistances (inverse of conductances) and capacitances separately (Kreindler et al., 2005; Singh et al., 2002).

**FIGURE 5:**
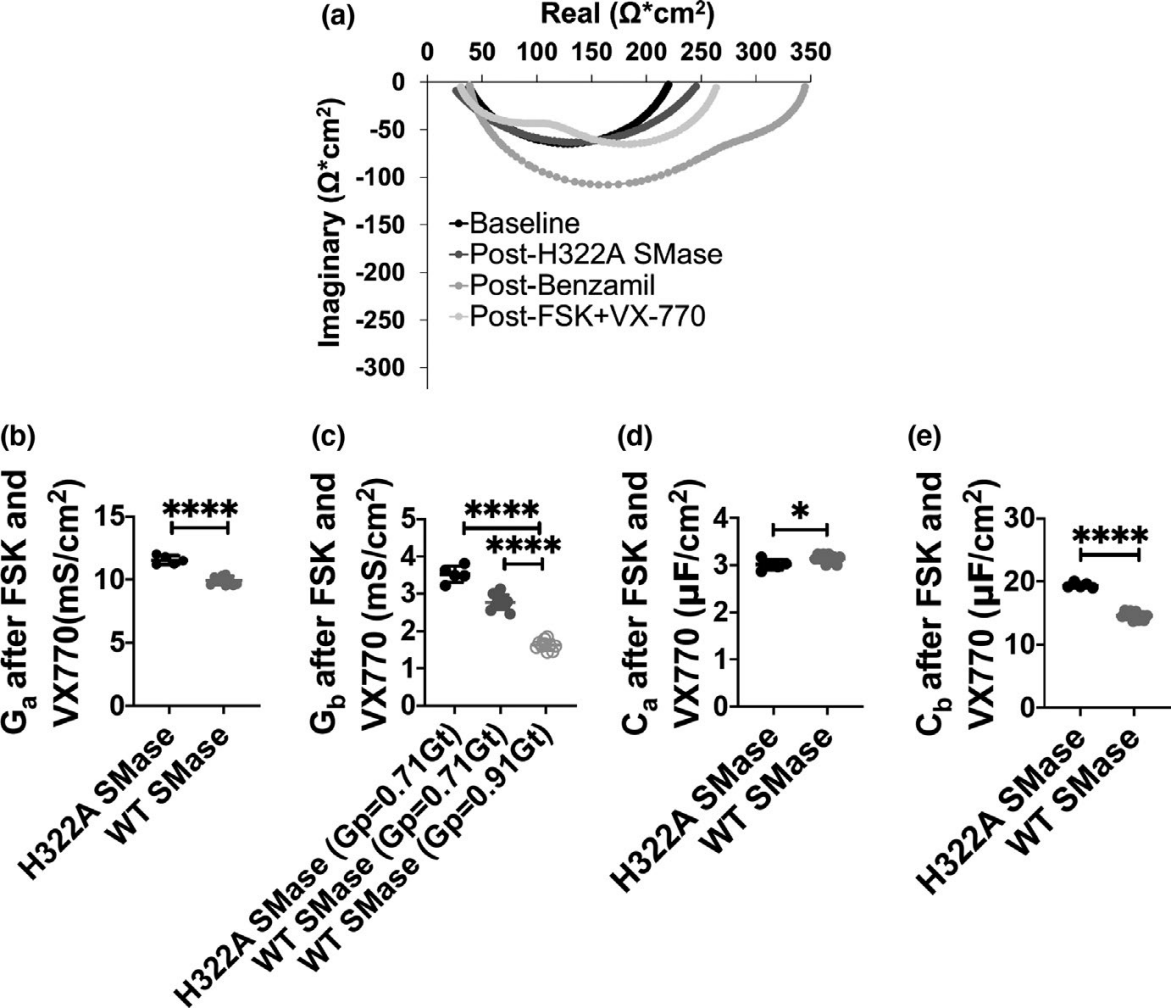
Impedance measurements of nHBEs (a single biological replicate) showed a reduction in both apical and basolateral conductance after WT SMase treatment, alongside a reduction of basolateral capacitance but an increase in apical capacitance. (a) A representative fit of the Nyquist Plot at baseline (black), after 30 min of 1 µg/ml enzyme‐dead H322A SMase basolaterally (dark gray), after at least 6 µM benzamil apically (medium gray), and after 10 µM forskolin and 1 µM VX770 (light gray) is shown. These fits were created by assuming that *G*_p_ is 71% of *G*_T_, and then iteratively determining the *R*_a_, *R*_b_, *C*_a_, and *C*_b_ that fit the primary data best. *R*_a_ and *R*_b_ were inverted to obtain the *G*_a_ and *G*_b_. (b) *G*_a_ and (c) *G*_b_ after forskolin and VX770 in cells‐treated basolaterally with either 1 µg/ml H322A (black, *n* = 5) or WT SMase (gray, *n* = 10) were analyzed by unpaired two‐tailed *t*‐tests. These analyses indicate that WT SMase significantly inhibited both *G*_a_ (****p <0.0001) and *G*_b_ (****p <0.0001) of nHBEs when assuming *G*_p_ = 0.71G_T_. Within *G*_b_, when assuming *G*_p_ = 0.91*G*_T_ (light gray, open circles), WT SMase appears to have an even more significant effect, with the *G*_b_ significantly decreased under this assumption as compared to the previous conditions (****p <0.0001). The (d) *C*_a_ and (e) *C*_b_ were analyzed by unpaired two‐tailed *t*‐tests. These analyses indicate that basolateral WT SMase significantly decreased *C*_b_ (****p <0.0001), while significantly increasing *C*_a_ (*p = 0.0322)

Impedance data were plotted as Nyquist plots (Figure 5a). The apical and basolateral resistances used to fit these Nyquist Plots were inverted into conductances, compiled, and analyzed (Figure 5b,c). WT SMase treatment decreased both the apical and basolateral conductance after addition of forskolin and VX770 in nHBEs. Forskolin‐ and VX770‐elicited apical conductance was reduced from 11.54 ± 0.35 to 9.93 ± 0.34 mS/cm^2^, an approximately 14% decrease. Basolateral conductance was reduced from 3.53 ± 0.22 to 2.77 ± 0.20 mS/cm^2^, an approximate 21% decrease. Since CFTR is the only apical channel activated after forskolin addition, this indicates that WT SMase decreases CFTR‐mediated transepithelial currents in part by decreasing the total conductance through apical CFTR. Furthermore, the basolateral membrane conductance data indicate that WT SMase additionally could be reducing CFTR‐mediated transepithelial currents by inhibiting a basolateral component of transepithelial flux, which is important for maintaining a driving force for apical anion secretion.

Along with conductance, the impedance analysis method also allowed for the determination of the apical and basolateral capacitances. Basolateral WT SMase decreased basolateral capacitance from 19.4 ± 0.42 to 14.5 ± 0.74 µF/cm^2^, an approximate 25% decrease. WT SMase also increased apical capacitance from 3.01 ± 0.11 to 3.10 ± 0.15 µF/cm^2^, an approximate 4% increase (Figure 5d,e).

### 3.5 WT SMase does not affect paracellular permeability in nHBEs

Part of the procedure to fit impedance data requires knowledge of the relationship between the paracellular and transepithelial conductances. While the value for this assumption does not greatly affect most of the calculated values, it can dramatically affect the value for the basolateral conductance (*G*_b_) (Singh et al., 2002). Thus, we needed to confirm that the assumed value for the relationship between paracellular and transepithelial conductance (*G*_p_, *G*_T_) was warranted following WT SMase treatment.

The assumed value relating *G*_p_ and *G*_T_ was based on prior experiments on nHBEs not treated with WT SMase, which indicated that *G*_p_ = 0.71*G*_T_ (unpublished observations). *G*_T_ is equal to the sum of *G*_p_ and transcellular conductance (*G*_c_). Thus, it is clear that the relationship between *G*_p_ and *G*_T_ can be affected by changing *G*_p_, *G*_c_, or both. WT SMase treatment does decrease the *G*_c_ (at least through the apical membrane, Figure 5b), which would increase the relative contribution of *G*_p_ to *G*_T_, assuming that *G*_p_ remained unchanged after WT SMase treatment. Fitting the WT SMase‐treated cells with a higher relative contribution of *G*_p_ (a lower relative contribution of *R*_p_) only makes the observed decrease in *G*_b_ after WT SMase treatment more extreme than when assuming the same *G*_p_ for both treatments (Figure 5c, gray open circles, 54% decrease in *G*_b_). Thus, we have biased our results toward the null by assuming *G*_p_ = 0.71*G*_T_. However, this still assumes that *G*_p_ remains unchanged by WT SMase treatment. While there is some evidence that 0.1 µM (but not 1 µM) ceramide treatment for 24 h may slightly decrease the permeability of tight junctions in H441 airway cells (Kalsi et al., 2019), we needed to determine whether WT SMase affected paracellular conductance under the conditions used here.

**FIGURE 6:**
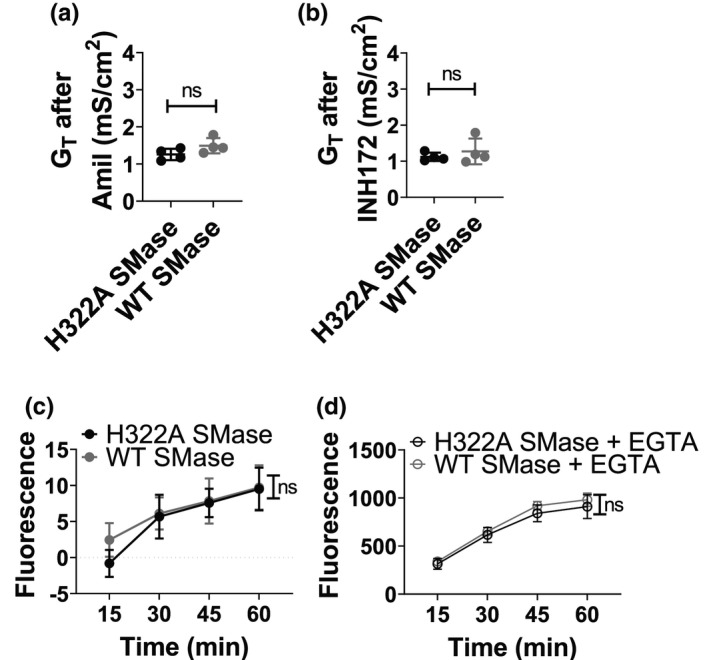
SMase does not affect baseline transepithelial conductance or calcein flux. Transepithelial conductances of nHBEs (a single biological replicate) were measured by Ussing Chamber under symmetric chloride conditions. Cells were allowed to stabilize for 30 min, after which 1 µg/ml enzyme‐dead H322A (black, *n* = 4) or WT (gray, *n* = 4) SMase was added to the basolateral side of the cells for 30 min. After this, 20 µM amiloride was added to the apical side, followed by 10 µM forskolin added to both sides. After the forskolin‐elicited current stabilized, 10 µM INH172 was added to the apical side to inhibit CFTR specifically. The conductances after (a) 20 µM amiloride and (b) 10 µM INH172 apically are shown. Data were analyzed by unpaired two‐tailed *t*‐tests, which indicate that WT SMase did not affect post‐amiloride or post‐INH172 conductances (*p* = 0.1171, *p* = 0.4561). (c) Paracellular flux of calcein in nHBEs was determined as described in methods. The assay involved adding 1 µg/ml H322A (black, *n* = 7) or WT (gray, *n* = 6–7) SMase basolaterally and 20 µg/ml calcein apically. (d) To determine flux under maximal paracellular flux conditions, tight junctions were disrupted with 3 mM EGTA. These EGTA‐treated cells were secondarily treated with either H322A (open black, *n* = 4) or WT (open gray, *n* = 4) SMase. Data were analyzed by a two‐way ANOVA for the EGTA treatment, and a mixed effects analysis for the no‐EGTA treatment due to missing values. In both cases, there were repeated measures over time. These analyses indicate no SMase‐induced differences in the transepithelial flux of calcein under normal and EGTA conditions (*F* = 0.4586, *p* = 0.2983), and that an increase in paracellular flux could have been detected

Under a chloride gradient, we found that there was no difference in the post‐amiloride or post‐INH172 short‐circuit currents, which are predominantly (not exclusively) controlled by paracellular ion movements since ENaC and CFTR are both inhibited (or inactivated) at both points (Figure S4a,d). These data suggest that WT SMase does not affect paracellular chloride conductance. However, tight junctions in bronchial epithelia show differential ion selectivity (Flynn et al., 2009). Therefore, to evaluate the paracellular conductance of all ions, the transepithelial conductance in the absence of an ion gradient was evaluated (normal chloride buffer on both sides). These data indicated no difference in the post‐amiloride or post‐INH172 conductance after treatment with SMase (Figure 6a,b). Thus, these data support the hypothesis that SMase does not affect paracellular conductance of any ion under the treatments explored in these experiments.

To further determine if paracellular tight junctions were affected by WT SMase, a calcein flux permeability assay was performed (Schlingmann et al., 2016; Stewart et al., 2017). WT SMase treatment over the course of 1 h did not affect calcein permeability (Figure 6c). To evaluate how these fluxes compared to those under conditions facilitating maximal paracellular flux, we treated one group of Transwells with 3 mM EGTA to allow unencumbered paracellular movement through tight junctions (Figure 6d). This group of wells was further subdivided into samples treated with either H322A or WT SMase. Treatment with 3 mM EGTA allowed for an approximately 100‐fold increase in the flux of calcein through the nHBE monolayers, and WT SMase treatment did not affect this, indicating that an increase in flux from the untreated cells (without EGTA) would have been detectable. We also confirmed via lipidomics analysis of Calu‐3 bronchial epithelial cells that WT SMase was still active in the presence of 3 mM EGTA in KRH (Figure S6). These results indicate that, over the time course used, WT SMase did not affect paracellular permeability. As such, our assumptions for fitting impedance data are supported, and the conclusion that WT SMase inhibits apical and basolateral membrane conductance in nHBEs can be maintained.

### 3.6 WT SMase inhibits CFTR current and conductance in cfHBEs

In order to determine if WT SMase similarly inhibited CFTR‐mediated transepithelial current and conductance in cells from people with CF, and to determine if current FDA‐approved CFTR modulators could overcome WT SMase‐mediated inhibition of CFTR, these experiments were repeated on cfHBEs homozygous for the ΔF508‐CFTR mutation. The ΔF508 mutation results in an improperly folded CFTR protein that does not traffic effectively to the cell surface. Trafficking of ΔF508‐CFTR to the cell membrane can be rescued (corrected) by temperature shift to 27°C; by treatment with FDA‐approved correctors such as VX809 (Lumacaftor) (Van Goor et al., 2011), VX661 (Tezacaftor), or VX445 (Elexacaftor) (Center for Drug Evaluation and Research, 2019; Keating et al., 2018); or by a combination of these drugs. Once at the surface, ΔF508‐CFTR still has difficulty in opening, and requires potentiators such as VX770 (Ivacaftor) to elicit more current. We have shown previously that WT SMase inhibits transepithelial currents in cfHBEs corrected by temperature shift alone (Stauffer et al., 2017), but have not determined if correction with VX809 or VX445/VX661 can prevent WT SMase‐mediated inhibition of CFTR‐mediated transepithelial currents in cfHBEs.

We first confirmed that WT SMase affected sphingolipids similarly in VX809‐corrected cfHBEs as in nHBEs (Figure S7a–d). Then, we performed short‐circuit current experiments on cfHBEs corrected with 3 µM VX809 and temperature shift to 27°C for 24 h (Figure S7e–g). Correction significantly reduced the amount of amiloride‐sensitive ENaC current in cfHBEs (Figure S7e). This makes sense, given that it is known that ENaC is overactive in CF cells as compared to non‐CF cells (Frizzell & Hanrahan, 2012). Interestingly, WT SMase treatment did not affect the ENaC currents in these corrected cells, contrary to in nHBEs (Figure S7e). VX809‐ and temperature‐shift correction did not rescue forskolin‐elicited currents alone (data not shown), but did rescue VX770‐potentiated and INH172‐sensitive currents (Figure S7f,g). WT SMase decreased the VX770‐potentiated and INH172‐sensitive currents in corrected cfHBEs (Figure S7f,g).

**FIGURE 7:**
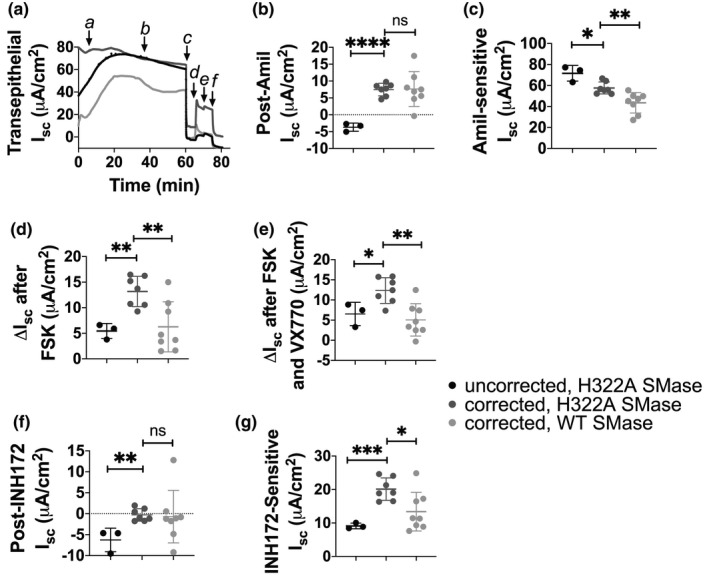
Transepithelial currents of cfHBEs (single biological replicate), either uncorrected (black) or corrected with 5 µM VX445 and 18 µM VX661 for 48 h (gray), were measured using an Ussing Chamber. (a) An example trace is shown. At the beginning of the experiment, (a) the correctors or vehicle were added to the appropriate cells. The cfHBEs were stabilized for 30 min, at which point (b) 1 µg/ml enzyme‐dead H322A (uncorrected, black, *n* = 4; corrected, dark gray, *n* = 5) or WT SMase (corrected, light gray, *n* = 4) was added basolaterally. After 30 min, (c) 20 µM amiloride was added apically, then (d) 10 µM forskolin was added. These forskolin‐elicited CFTR currents were potentiated by addition of (e) 1 µM VX770. Finally, CFTR currents were specifically inhibited by apical addition of (f) 10 µM INH172. All currents were analyzed by unpaired two‐tailed *t*‐tests. Analysis of the absolute currents after amiloride (b) and after INH172 (f) indicate that VX445/VX661‐mediated correction of cfHBEs significantly increased these baseline currents (****p <0.0001, **p = 0.0019). However, WT SMase did not affect these baseline currents in the corrected cfHBEs (*p* = 0.9423, *p* = 0.8678). (c) Analysis of the amiloride‐sensitive current indicates that correction significantly reduced ENaC currents (*p = 0.0114). WT SMase further decreased these ENaC currents in VX445/VX661‐corrected cfHBEs (**p = 0.0052). (d) Analysis of the changes in current from the post‐amiloride current to the post‐forskolin current indicates that correction significantly rescued forskolin‐elicited currents (**p = 0.0030). WT SMase significantly decreased these currents (**p = 0.0064). (e) Analysis of the changes in current from the post‐amiloride current to the post‐VX770 current indicates that correction significantly rescued VX770‐potentiated currents (*p = 0.0274). However, WT SMase significantly decreased these currents (**p = 0.0020). (g) Analysis of the changes in current from the post‐VX770 current to the post‐INH172 current indicates that correction significantly rescued INH172‐sensitive currents (***p = 0.0006). However, WT SMase significantly decreased these currents (*p = 0.0183). Altogether, this suggests that correction was effective, but that WT SMase still inhibited CFTR‐mediated currents in VX445/VX661‐corrected cfHBEs

The more clinically relevant drugs in the current Trikafta^®^ therapy, VX445, and VX661, were evaluated as well. In this case, cfHBEs were treated with 5 µM VX445 and 18 µM VX661 for 48 h (Figure 7). As in the VX809‐ and temperature‐corrected cfHBEs, VX445/VX661 correction decreased amiloride‐sensitive ENaC currents (Figure 7c). However, this may have been in part due to an elevated post‐amiloride baseline current in these corrected cells (Figure 7b). This elevated baseline remained even after INH172 addition at the end of the experiment, indicating that it was not CFTR current (Figure 7f). The origin of this elevated baseline current has not yet been explored. Corrected VX445/VX661‐corrected cfHBEs also had more forskolin‐elicited, VX770‐potentiated, and INH172‐sensitive current than their uncorrected counterparts (Figure 7d,e,g). The corrected cfHBE cells treated with H322A SMase were then compared to corrected cfHBE cells treated with WT SMase. In cfHBEs corrected with VX445/VX661, WT SMase decreased the forskolin‐elicited, VX770‐potentiated, and INH172‐sensitive CFTR currents from 13.17 ± 2.96 to 6.26 ± 1.86 µA/cm^2^, from 12.34 ± 2.90 to 5.06 ± 4.01 µA/cm^2^, and from 20.11 ± 3.35 to 13.39 ± 5.78 µA/cm^2^, respectively. These reductions are similar to those seen in nHBEs, and again indicate that WT SMase reduces CFTR‐mediated transepithelial currents in cfHBEs corrected with current FDA‐approved CFTR modulators.

**FIGURE 8:**
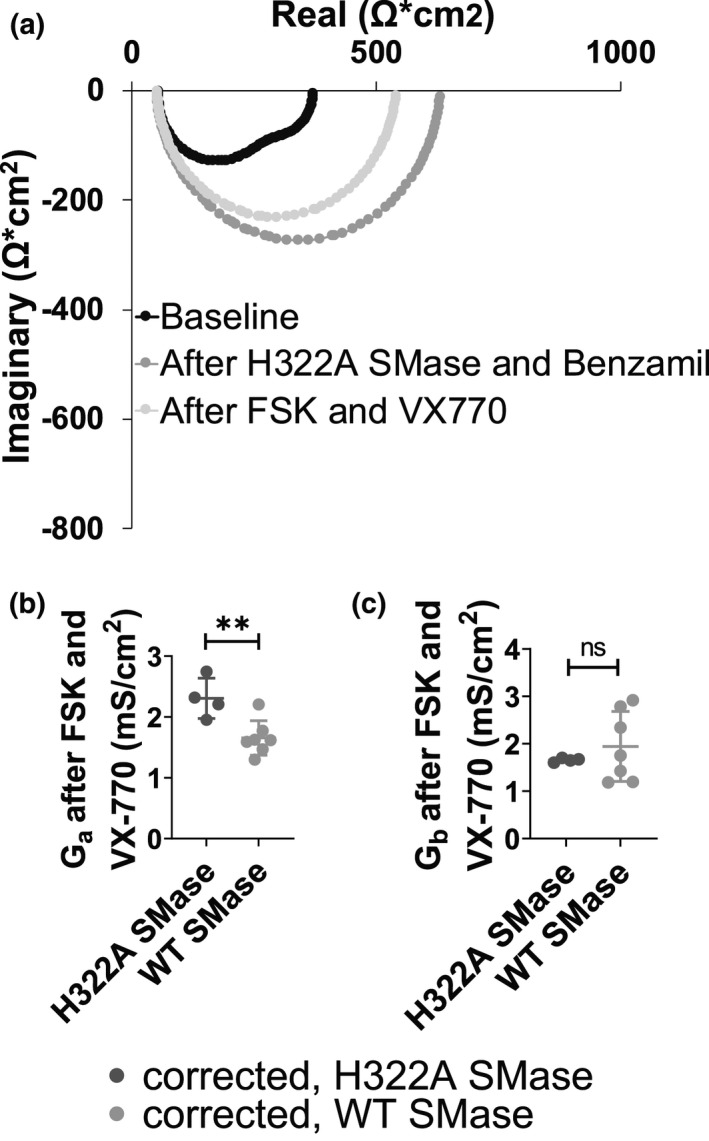
Apical conductance (*G*_a_) was decreased but basolateral conductance (*G*_b_) was unaffected by WT SMase in C18‐corrected cfHBEs (a single biological replicate), as determined by impedance analysis. (a) A representative Nyquist Plot is shown of cfHBEs corrected with 6 µM C18 for 24 h at baseline (black), after 30 min of 1 µg/ml enzyme‐dead H322A SMase basolaterally and 6 µM benzamil apically (medium gray), and after 10 µM forskolin and 1 µM VX770 (light gray). The (b) *G*_a_ and (c) *G*_b_ after forskolin and VX770 in these C18‐corrected cfHBEs‐treated basolaterally with either 1 µg/ml H322A (black, *n* = 4) or WT SMase (gray, *n* = 7) were analyzed by unpaired two‐tailed *t*‐tests. These analyses indicate that WT SMase significantly decreased *G*_a_ (**p = 0.0072), but did not affect *G*_b_ (*p* = 0.4369)

In continuing to compare the effects of WT SMase on cfHBEs to the effects on nHBEs, we used impedance analysis to determine the effects of WT SMase treatment on apical and basolateral membrane conductance in cfHBEs with the VX809 analog C18 (6 µM for 24 h). Note that at baseline, the Nyquist Plot of the corrected cfHBEs appeared as two distinct semi‐circles (Figure 8a, black). This phenomenon occurred in the uncorrected cfHBEs as well (data not shown), but did not occur in the nHBEs (Figure 5a, black). The presence of two semi‐circles at baseline in the corrected cfHBEs suggests that the apical and basolateral membrane have very different time constants. Furthermore, in corrected cfHBEs, the plots following forskolin and VX770 addition appear as only a single semi‐circle (Figure 8a, light gray). This is contrary to nHBEs, which did show two distinct semi‐circles following forskolin and VX770 (Figure 5a, light gray). Similar to the nHBEs, though, in C18‐corrected cfHBEs, apical conductance again was decreased by WT SMase from 2.31 ± 0.33 to 1.66 ± 0.28 mS/cm^2^, an approximately 28% decrease (Figure 8b, dark gray vs. light gray). This indicates that WT SMase inhibited CFTR‐mediated apical membrane conductance in cfHBEs corrected and potentiated by currently available FDA‐approved small molecule modulators. Interestingly, though, the mean of the basolateral conductance was not affected by WT SMase, even though the variability increased dramatically relative to the control (Figure 8c). This does not match what was seen in nHBEs, where conductance at both membranes was inhibited. More work is necessary to understand this discrepancy.

## 4 DISCUSSION

The data presented here indicate that acute basolateral exposure to WT SMase decreases transepithelial CFTR currents in nHBEs, nHTEs, and chemically corrected cfHBEs. Furthermore, as found previously (Stauffer et al., 2017), VX770 did not recover currents in nHBEs or corrected cfHBEs treated with WT SMase. It remains to be determined if these reduced currents were due to decreased secretion of chloride, bicarbonate, or both. Note that we distinguish between a reduction of current (indicative of the net vectorial movement of ions) and an inhibition of membrane conductance (indicative of the potential for total movement of ions in both directions). Reduced transepithelial currents can be caused by reduced apical membrane conductance, reduced basolateral membrane conductance, reduced paracellular conductance, or any combination of these.

We determined in this study that WT SMase does not affect paracellular conductance, leaving only apical and/or basolateral conductance as the likely causes of changes to transepithelial current. While previous work in Calu‐3s attempted to isolate the apical and basolateral membrane conductances by utilizing the pore‐forming antifungal nystatin (Ito et al., 2004), we specifically chose not to use this technique. Since both WT SMase and nystatin perturb lipid rafts, with WT SMase disrupting sphingomyelin and nystatin disrupting cholesterol, the use of both could cause competition between them (Abu‐Arish et al., 2019; Coutinho et al., 2004). Instead, we utilized the nondestructive impedance analysis method to study intact epithelia.

Using impedance analysis, we found that WT SMase decreased transepithelial CFTR currents in part by inhibiting CFTR‐mediated apical membrane conductance in nHBEs and corrected cfHBEs. Furthermore, in nHBEs, impedance analysis revealed that WT SMase decreased basolateral membrane conductance. This decreased basolateral conductance was likely due to decreased activity of potassium channels, which contribute to transepithelial anion secretion by repolarizing the membrane potential thereby driving anions out of the cell across the apical membrane. Thus, in future experiments to understand the mechanism of WT SMase‐mediated inhibition of CFTR current in nHBEs, it will be important to perform impedance analysis experiments to distinguish effects at the apical versus the basolateral membrane. Interestingly, though, decreased basolateral conductance was not seen in cfHBEs. These results suggest that WT SMase acts on both apical and basal membrane conductance in nHBEs but only the apical membrane conductance of corrected cfHBEs. The reason for this discrepancy remains to be explored.

In apparent contradiction to our findings, recently it was found that treating ${\text{CFBE41o}}^{‐}$ and nHBE cells acutely with the acid‐SMase inhibitor amitriptyline reduced sequential vasoactive intestinal peptide (VIP) and forskolin‐elicited transepithelial currents (Abu‐Arish et al., 2019). Abu‐Arish et al. (2019) found that VIP stimulation resulted in an acid‐SMase‐dependent increase in ceramide, which formed platforms where CFTR clustered. Furthermore, VIP stimulation caused an increase in surface expression of CFTR. Acute amitriptyline treatment, without affecting the surface expression of acid‐SMase, prevented both the acid‐SMase‐dependent generation of ceramide and the increased surface expression of CFTR caused by VIP stimulation (Abu‐Arish et al., 2019). While this appears to conflict with our results obtained with bacterial WT SMase, these authors did not evaluate forskolin treatment independently of VIP treatment, which is what we evaluated here. Furthermore, amitriptyline causes a functional inhibition of acid‐SMase by accumulating in the lysosome and inducing dissociation of acid‐SMase from the lysosomal membrane, resulting in its proteolysis (Kornhuber et al., 2008). Amitriptyline is not specific to acid‐SMase in this mechanism of lysosomal membrane dissociation and subsequent proteolysis (Elojeimy et al., 2006; Kolzer et al., 2004; Zeidan et al., 2006), and thus likely has off target effects that need to be considered. Furthermore, it does not make mechanistic sense for amitriptyline to have affected surface acid SMase activity without affecting its expression, again, since amitriptyline causes the degradation of acid SMase in the lysosome, not at the surface of the cell.

Given the data presented thus far, it is relevant to determine the mechanism of WT SMase‐mediated decreases in CFTR activity in HBEs. Our observed lack of acute‐VX770‐mediated recovery in cells treated with WT SMase does give some insight into the mechanism of inhibition. The fact that VX770 does not rescue the decreased forskolin‐elicited or INH172‐sensitive current or conductance indicates that WT SMase does not reduce CFTR‐mediated currents and conductances by reducing PKA‐mediated phosphorylation. As we showed previously in nHBEs, a reduced level of PKA phosphorylation allows for greater VX770‐mediated potentiation of CFTR‐mediated currents, not less (Cui et al., 2019). Thus, if WT SMase was inhibiting CFTR activity simply by decreasing PKA phosphorylation, VX770 would have recovered the CFTR‐mediated current and conductance. Similarly, we previously showed that WT SMase inhibits CFTR channels made independent of PKA‐mediated phosphorylation by removal of the regulatory (R‐) domain—which bears the sites phosphorylated by PKA—when these channels are expressed in *Xenopus* oocytes (Stauffer et al., 2017). A mechanism unrelated to PKA‐mediated phosphorylation must be involved, and this mechanism must be determined to develop potential therapeutic strategies to negate WT SMase‐mediated decreases in CFTR currents in bronchial epithelial cells.

It is also important to determine the clinical relevance of the effects of WT SMase on these cells. This includes determining the imbalance of sphingolipids as well as sphingolipid modulating enzymes in cfHBEs compared to nHBEs. Recent evidence from multiple laboratories using multiple methods, including thin layer chromatography and mass spectrometry, has indicated that ceramides are increased in polarized bronchial epithelial cells from people living with CF (homozygous or heterozygous for the ΔF508‐CFTR mutation), as compared to cells from non‐CF subjects (Gardner et al., 2020; Loberto et al., 2020). One of these papers suggested that this imbalance could be due to decreased activity of acid‐ceramidase, though increased acid‐SMase activity was also observed (Gardner et al., 2020). Importantly however, conflicting data also has been found in which overall ceramide levels are actually decreased in CF cells compared to non‐CF cells, though the ratio of long‐chain ceramides to very‐long‐chain ceramides is increased (Veltman et al., 2021). Due to this conflicting data, it will be important for other groups to evaluate sphingolipid imbalances in cfHBEs compared to nHBEs.

## 5 CONCLUSION

We have shown that the bacterial virulence factor WT SMase causes a reduction of CFTR current in nHBEs and nHTEs, and in cfHBEs corrected with Tezacaftor/Elexacaftor or Lumacaftor. This WT SMase exposure was also associated with an indiscriminate conversion of sphingomyelins into ceramides. The loss of current is not recoverable by Ivacaftor (VX770) treatment, underscoring the clinical importance of this finding. We then determined by impedance analysis that basolaterally applied SMase caused an inhibition of apically located CFTR in both nHBEs and corrected cfHBEs. We also found via calcein flux assays that SMase did not affect paracellular permeability within the context of these experiments. This is clinically relevant, since ceramide levels are elevated in many disease states, including CF, and since acid‐SMase is released endogenously upon inflammatory stimulation. Further work will determine the effects of long‐term SMase treatment and the exact mechanism by which SMase inhibits CFTR.

## CONFLICT OF INTEREST

The authors have no conflicts of interest to report.

## AUTHOR CONTRIBUTION

KAC performed experiments and wrote the manuscript. RJP and MK provided expertise on tight junctions and calcein flux assays. MK provided bronchial epithelial cells for study and expertise on sphingolipids. CFL provided expertise on impedance analysis. RJB provided expertise, equipment, and reagents (including bronchial epithelial cells) for impedance analysis, and provided expertise on CFTR and bronchial epithelial cells. NAM provided funding as well as expertise on CF, CFTR, Ussing Chamber, and bronchial epithelial cells.

## Supporting information



Figure S1–S7

## Data Availability

Data are available upon request from N.A.M. (namccar@emory.edu).
