# Supplementary material for: Sphingomyelinase decreases transepithelial anion secretion in airway epithelial cells in part by inhibiting CFTR‐mediated apical conductance
Source: Physiol Rep. 2021 Aug 12;9(15):e14928. doi: 10.14814/phy2.14928 (PMC8358481; doi:10.14814/phy2.14928)
Supplement: Supplementary file 1 — Figure S1–S7 [file PHY2-9-e14928-s001.pdf]

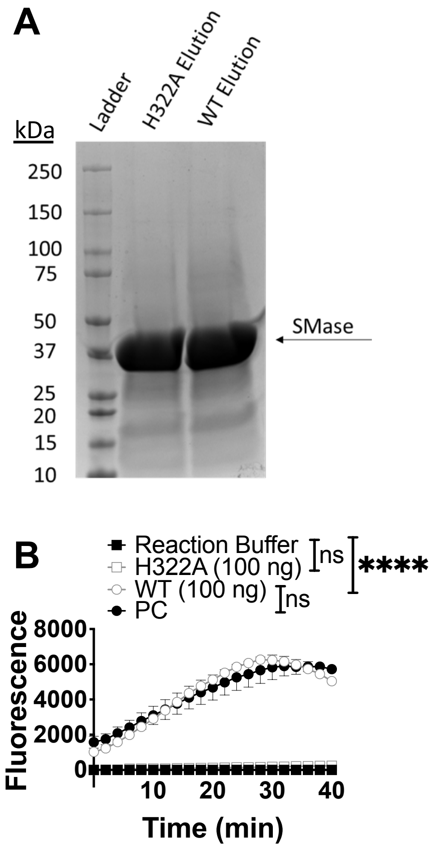

Figure S1. (A) H322A or WT SMase purified as described in Methods was intentionally overloaded for an SDS-PAGE experiment to show impurities. Total protein in the gel was stained with Coomassie blue. SMase (37 kD) is the most prominent band by far, but there are other co-eluted proteins. **(B)** SMase activity was assessed by an Amplex Red assay as described in Methods. The generation of fluorescent product over time was compared between groups by multiple two-way ANOVAs. H322A SMase (open square, n= 4) had no detectible activity above the reaction buffer control (solid square, n=4) (p=0.0718), while WT SMase activity (open circle, n=4) was very easily detected over the reaction buffer (****p<0.0001), and WT SMase activity was not distinguishable from the phosphocholine positive control group (solid circle, n=4) (p=0.8478).

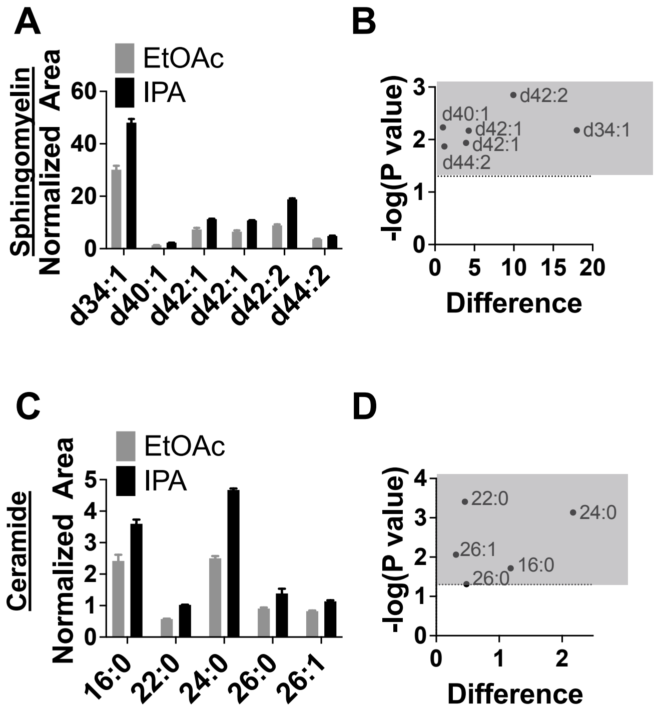

Figure S2. Lipidomics mass spectrometry results of **(A,B)** sphingomyelins and **(C,D)** ceramides found in Calu-3 bronchial epithelial cells treated basolaterally with 1 μg/mL enzyme-dead H322A SMase indicated that isopropyl alcohol (IPA) extraction was more efficient than ethyl acetate (EtOAc) extraction. Sphingolipids either were extracted with the IPA method described in Methods (black, n=2), or with EtOAc extraction described previously (grey, n=2).[1,2] **(A,C)** The peak amplitudes were normalized as described in Methods. Data were analyzed by multiple *t* tests with a 5% false discovery rate. **(B, D)** Volcano plots of the differences between the H322A and WT SMase treatments, with the –log(P value) on the y-axis and the difference between the averages of the normalized peak areas on the x-axis are shown. The horizontal dotted line (-log(P value)=1.30) indicates the significance cutoff, above which a species is considered significantly different. This significance area is highlighted with a dark grey box All sphingomyelins and ceramides of interest were significantly more abundant when extracted with IPA.

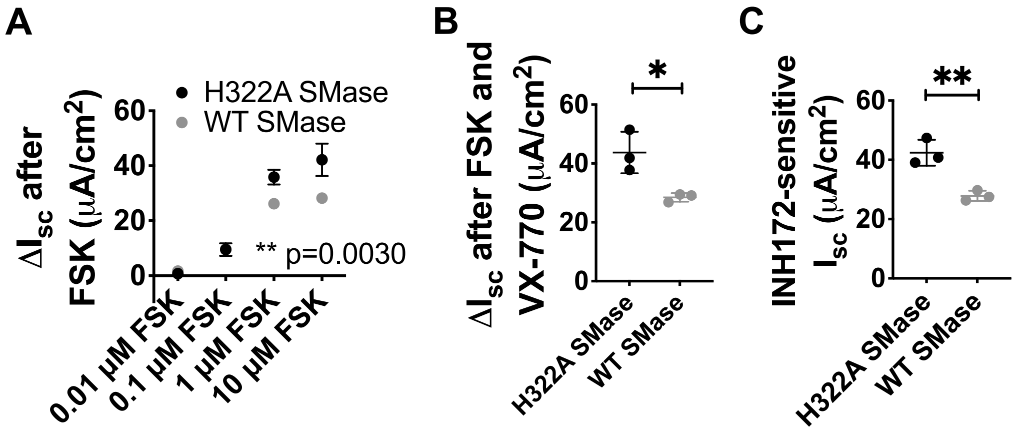

Figure S3. A reduction in CFTR short-circuit currents was seen in non-CF human tracheal epithelial cells (nHTEs) treated basolaterally with 1 µg/mL WT SMase (grey, n=3) as compared to enzyme-dead H322A SMase (black, n=3) when measured using an Ussing Chamber. **(A)** The absolute changes in current from the post-amiloride current to the current elicited by various concentrations of forskolin were analyzed by a two-way ANOVA with repeated measures over the concentrations of forskolin. This analysis indicates that SMase significantly decreased the forskolin-elicited currents in nHTEs (**p=0.0030). The absolute changes in current **(B)** from the post-amiloride current to the post-VX-770 current and **(C)** from the post-VX-770 current to the post-INH172 current were analyzed by unpaired two-tailed *t* tests. These analyses indicate that SMase significantly decreased the **(B)** VX-770-potentiated current (*p=0.0213) and **(C)** INH172-sensitive current (**p=0.0058) in nHTEs.

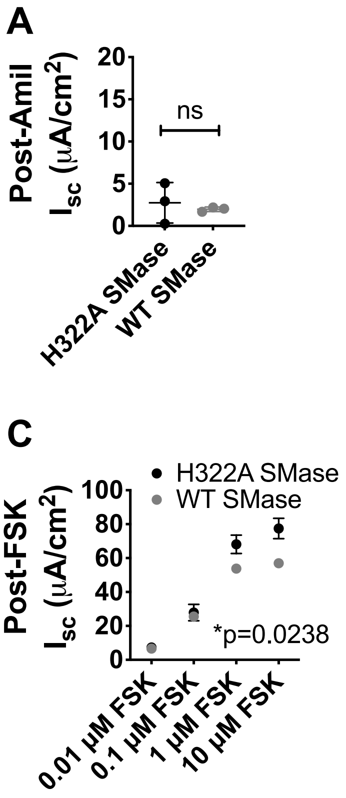

Figure S4. Short-circuit currents from nHBEs treated basolaterally either with 1 µg/mL enzyme-dead H322A (black, n=3) or WT SMase (grey, n=3) were measured using an Ussing Chamber system. The experimental setup was that of Figure 3. **(A)** The absolute current after 20 µM amiloride, **(B)** the change from the pre- to the post-amiloride current, and **(D)** the current after 10 µM INH172 were analyzed by unpaired two-tailed *t* tests. These analyses indicate that SMase **(A)** did not affect the post-amiloride baseline current (p=0.6087), **(B)** significantly inhibited amiloride-sensitive currents (**p=0.0074), and **(D)** did not affect the absolute post-INH172 current (p=0.1953). **(C)** The currents after various concentrations of forskolin, plotted as the means and standard deviations, were analyzed by a two-way ANOVA with repeated measures over the concentrations of forskolin. This analysis indicates that SMase significantly decreased the absolute currents after forskolin (*p=0.0238).

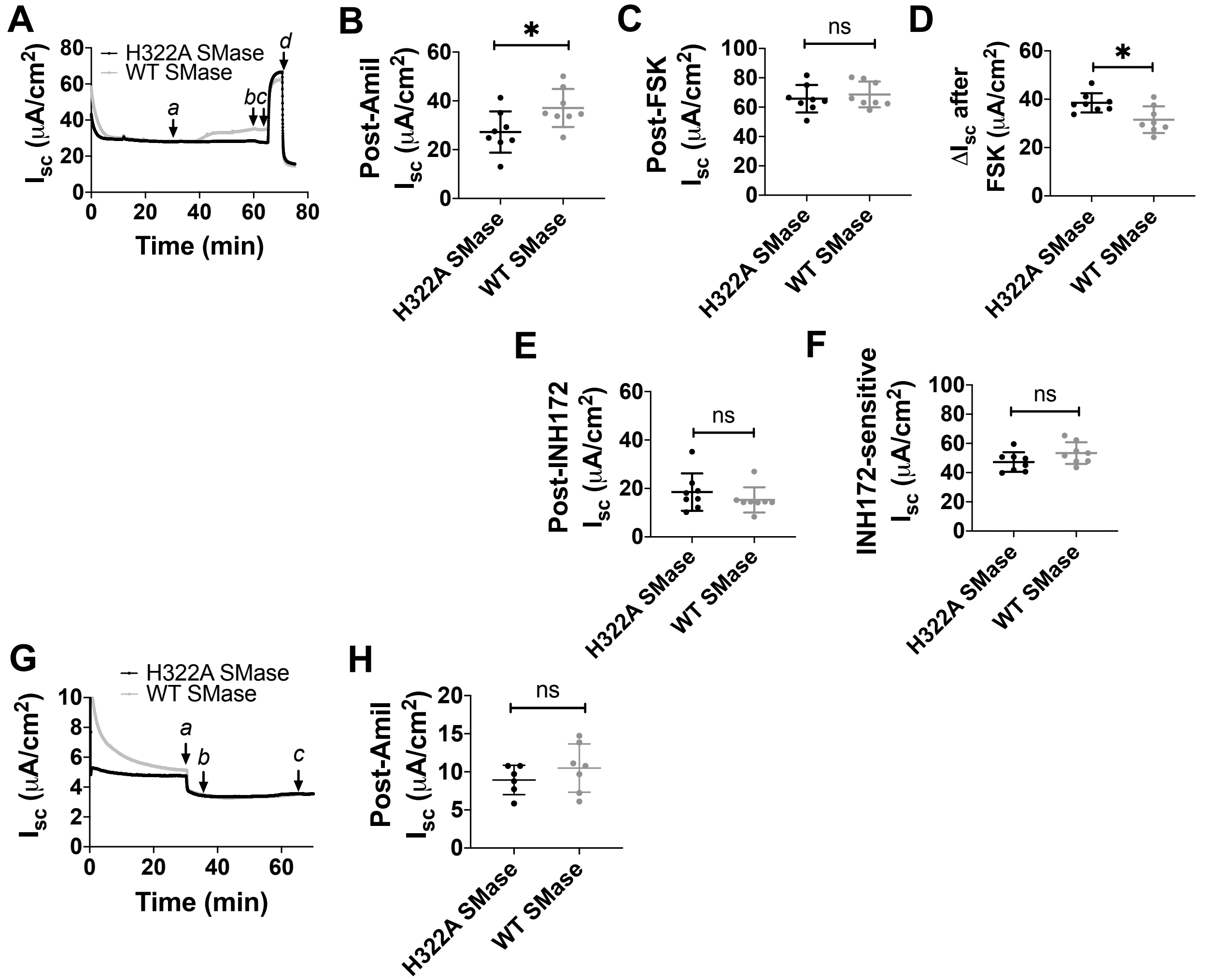

Figure S5. Short-circuit transepithelial currents of 16HBEs were measured using an Ussing Chamber. **(A)** An example trace is shown. Cells were stabilized for 30 min, at which point *(a)* 0.1 μg/mL enzyme-dead H322A (black, n=8) or WT SMase (grey, n=8) was added basolaterally. After 30 min, *(b)* 20 μM amiloride was added apically, then *(c)* 10 μM forskolin was added. These forskolin-elicited CFTR currents were inhibited by *(d)* apical addition of 10 μM INH172. All analyses were by unpaired two-tailed *t* tests. **(B)** The absolute currents after amiloride were significantly increased in the SMase-treated group as compared to the H322A SMase control (*p=0.0296). The amiloride-sensitive currents were very close to zero, and thus were not analyzed. **(C)** The absolute current after forskolin was not affected by SMase (p=0.5359) but **(D)** the changes in current from the post-amiloride current to this current were significantly decreased by SMase (*p=0.0116). **(E)** The absolute currents after INH172 and **(F)** the changes in current from the post-forskolin current to this current were not affected by SMase (p=0.3437, p=0.1065). Taken together, these data suggest that in 16HBEs, SMase activated CFTR prior to forskolin addition. **(G)** Experiments were conducted in which CFTR was inhibited prior to SMase treatment, and an example trace is shown. Cells were stabilized for 30 min, at which point *(a)* 10 μM INH172 was added apically. Then *(b)* 0.2 μg/mL enzyme-dead H322A (black, n=6) or WT SMase (grey, n=7) was added basolaterally. After 30 min, *(c)* 20 μM amiloride was added apically to inhibit ENaC currents. When CFTR was already inhibited, **(H)** the absolute currents after amiloride were not increased by SMase (p=0.3154).

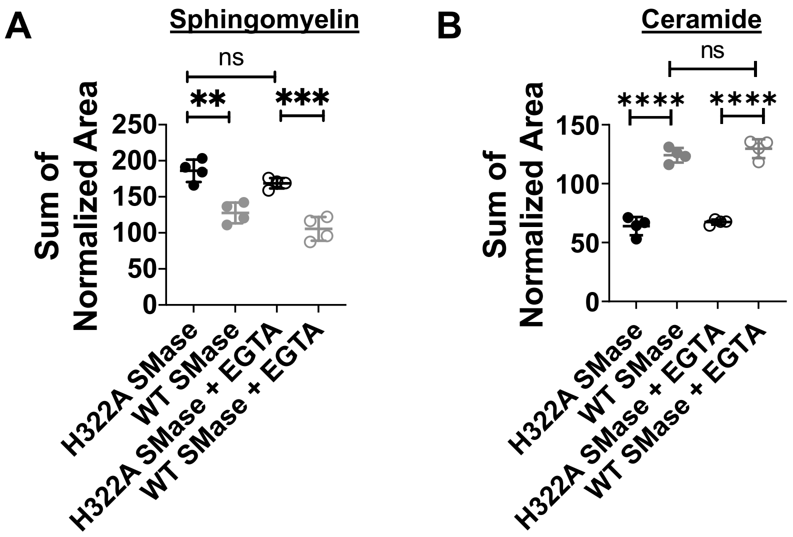

Figure S6. Mass spectrometry analysis of (A) sphingomyelins and (B) ceramides in Calu-3 cells treated as in the calcein flux experiments (Figure 6) indicated that in the presence of 3 mM EGTA in KRH buffer, WT SMase retained its ability to hydrolyze sphingomyelin into ceramide. The normalized area of all sphingomyelins or ceramides were added together and reported here. Two-way ANOVAs with multiple comparisons and Tukey correction were used to analyze the data. As seen previously under control conditions, WT SMase (closed grey circles, n=4) decreased sphingomyelins and increased ceramides relative to H322A SMase (closed black circles, n=4) (***p=0.0003; ****p<0.0001). Similarly, in 3 mM EGTA conditions, WT SMase (open grey circles, n=4) decreased sphingomyelins and increased ceramides relative to H322A SMase (open black circles, n=4) (***p=0.0002; ****p<0.0001). There was no significant difference in sum of the normalized area of sphingomyelins or ceramides in cells treated with WT SMase, regardless of EGTA treatment (p=0.1644; p=0.6282).

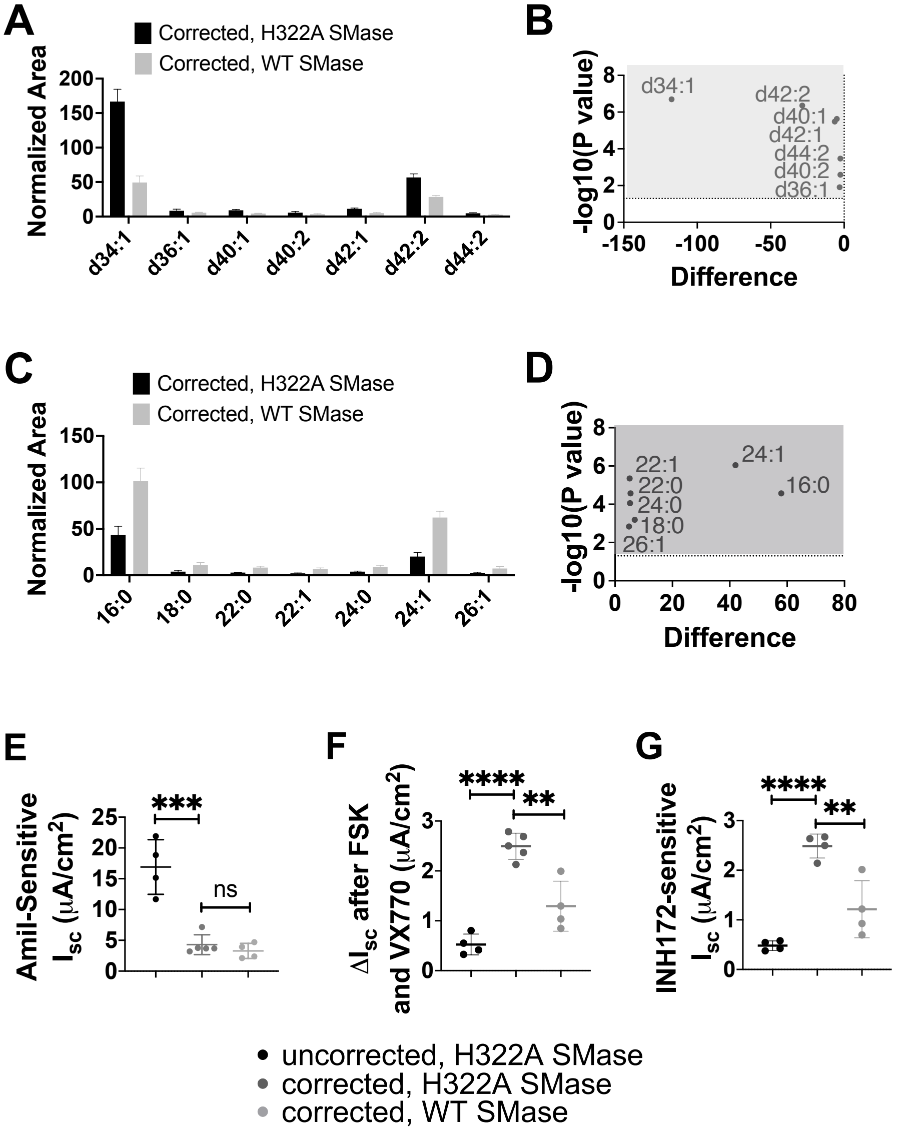

Figure S7. In VX809-corrected cfHBEs, SMase (grey, n=6) **(A,B)** decreased all sphingomyelins and **(C,D)** increased all ceramides of interest compared to enzyme-dead H322A SMase control (black, n=5). **(E-G)** SMase treatment of cfHBEs either uncorrected (black, n=4) or corrected (grey) with 3 μM VX-809 and temperature shift to 27 °C for 24 hr showed changes in short-circuit currents, as measured by an Ussing Chamber in symmetric chloride conditions. Basolaterally, 1 μg/mL enzyme-dead H322A (dark grey, n=5) or WT SMase (light grey, n=4) was added for 30 min. After this, 20 μM amiloride was added apically, followed by 10 μM forskolin, and then 1 μM VX-770. After this current stabilized, 10 μM INH172 was added apically. All data from these experiments were analyzed by unpaired two-tailed *t* tests. **(E)** Correction significantly decreased the amount of amiloride-sensitive ENaC current (***p=0.0006), and SMase did not further affect this (p=0.3357). Correction significantly increased the amount of **(F)** VX-770-potentiated (****p<0.0001) and **(G)** INH172-sensitive (****p<0.0001) currents in cfHBEs. SMase significantly decreased both the VX770-potentiated (**p=0.0023) and INH172-sensitive (**p=0.0065) currents. SMase decreased the maximal CFTR currents and INH172-sensitive currents to approximately 52% and 49% of the corrected control cells, respectively.
